# Close-range photogrammetry reveals morphometric changes on replicative ground stones

**DOI:** 10.1371/journal.pone.0289807

**Published:** 2023-08-22

**Authors:** Giusi Sorrentino, Fabio Menna, Fabio Remondino, Marco Paggi, Laura Longo, Alessandro Borghi, Alessandro Re, Alessandro Lo Giudice

**Affiliations:** 1 Department of Physics, University of Turin, Turin, Italy; 2 INFN, Section of Turin, Turin, Italy; 3 3D Optical Metrology Unit, Bruno Kessler Foundation (FBK), Trento, Italy; 4 IMT School for Advanced Studies Lucca, Lucca, Italy; 5 DAIS Department of Environmental Sciences, Ca’ Foscari University of Venice, Informatics and Statistics, Venice, Italy; 6 Department of Earth Science, University of Turin, Turin, Italy; The University of Tulsa, UNITED STATES

## Abstract

The pursuit of a quantitative approach to functional analysis of stone tools is an ongoing endeavour for traceologists. Technological advancements in 3D imaging techniques, such as photogrammetry/3D scanners, CT scanning, 3D digital microscopy, confocal microscopy, AFM and FEG-SEM and micro-topographical scanning, have greatly facilitated the detailed capturing of the geometry and surface texture at multiple levels of observation, from the object-scale to the nano-scale. However, while such technological innovations have predominantly focused on flaked assemblages, ground stone tools have only recently begun to receive due attention, and a standardised protocol for their study is yet to be established. In order to comprehend the function(s) of these tools, analytical techniques that enable a 3D visualisation of the entire item and the wear affecting the used surfaces have proven to be of great support. To this end, an analytical procedure was developed and tested on slabs and pebbles in order to replicate the use-wear traces observed on Upper Palaeolithic tools. The purpose was to assemble a site-specific reference collection tailored on the artefacts from the cultural level III of the Brînzeni I cave in north-west Moldova. Experimental replicas were used to treat different plant organs during controlled sequential experiments. The present article reports on the analysis based on photogrammetric data acquired during two stages of replicative usage. We tested multiple acquisition setups and elaborations to assess the geometry modification and the surface depletion. By exploring various acquisition strategies, a critical evaluation of potential sources of bias in data collection and subsequent elaboration were performed, and the methodology was accordingly adjusted thereby enhancing the reliability and reproducibility of the results. This study highlights the importance of carefully considering the acquisition strategy in archaeological related research to ensure accurate analyses and to validate robust interpretation.

## Introduction

Ground stones are a category of tools, made from pebbles and cobbles used in their natural form, or designed and manufactured for several different functions [1]. Their study in prehistoric archaeology is increasingly attracting attention for their significant value in providing insights into past societies, human habits and organisation, and even on the exploitation of the resources of a given environment. However, discerning their use, which involves gestures like hammering, battering, grinding, pounding various raw materials such as plants, ochre, skin, bones, etc., is not always straightforward. Identifying surface modifications, use-wear traces, and potential authentic residues requires a tailored approach and the application of different analytical techniques.

At the dawn of the discipline, the analysis of ground stone tools (GSTs) was conducted through microscopic observation and manual measurements (e.g., [2–6]). However, advancements in digital technology have presented new opportunities to analyse these artefacts in greater detail. Over the last decade, 3D stone artefact reconstruction has emerged as a prominent analytical method due to its versatility, precision, and wide range of potential applications, mostly used as pedagogical, illustrative, documentative instruments with a publication rate of over 200 articles from 2002 [7]. The use of diverse 3D scanning techniques such as laser, structured light, CT, and photogrammetry has facilitated the acquisition of volumetric data allowing three-dimensional virtual reconstruction of the complete artefact. These reconstructions are invaluable for scientists and archaeologists enabling a variety of analyses such as morphometric evaluations, development of specific texture description and quantification indices, and assessment of the operational chain (for an extensive review see [7–9]). The possibility to conduct quantitative analysis using 3D virtual replicas would be a significant accomplishment, particularly in the field of morphometric and functional analysis, where the need for unique unambiguous descriptions has long been at the forefront of researchers’ discussion. In the analysis of ground stone tools, having a digital model of the artefact is particularly valuable, as the studied items may be located in museums abroad and not available for direct inspection. A 3D model allows researchers to effectively investigate stones’ morphology, traces of use and technological aspects, for archaeological, ethnographic, or behavioural studies (i.e., on modern primates using stones) and experimentally produced/used tools (e.g., [10–23]). Also, it eases to exchange material in a digital format enhancing the communication and popularisation of analytical results.

The utilisation of 3D scanning and photogrammetry has become a standard practice in archaeological projects to meet the requirements of a documentation strategy less susceptible to the operator’s subjective choices and interpretations, such as the selection of the point of view to be displayed or the specific features to be represented in typical manual drawings. With 3D scanning and photogrammetry, a comprehensive full view of the object can be achieved, where the selection of features to be displayed is determined by technical resolution rather than the operator’s interpretation, enabling further analysis also by different researchers. It also allows the exchange of the digital data facilitating diverse interpretations of the archaeological findings. 3D scanners employ a light source, such as a laser or a structured pattern of light, along with cameras to record the light deformation [24]. Nonetheless, 3D scanning devices are often expensive and their portability may be limited making it difficult to acquire them or adopt them in suboptimal conditions. On the other hand, photogrammetry involves capturing sets of overlapping photographs taken from different angles and positions, identifying reference points on the images, and then using triangulation to determine the relative position of these points in 3D space [24,25]. Moreover, photogrammetry is a versatile and cost-effective technique that has become popular in cultural heritage studies due to its low equipment requirements—primarily a camera and photogrammetric software. Although photogrammetry may seem like a more user-friendly option compared to 3D scanning, which calls for technical knowledge before use, it is actually more engaging. Indeed, it requires more involvement and expertise from the operator to ensure high-quality results. In contrast, 3D scanning requires less intervention once the operator is trained. Therefore, whereas photogrammetry may seem more accessible, achieving accurate and reliable results with this technique requires mandatory adequate preparation and experience on the part of the operator. While the flexibility of photogrammetric methods and the abundance of free and low-cost commercial software make it an attractive option, there is currently a lack of standardisation in data acquisition protocols, eventually leading to unreliable metrical data. If it is true that photogrammetry can produce visually impressive outcomes, suitable for documentation and illustrative purposes, it is crucial to exercise caution when considering analytical needs. According to Barone [23,26] accuracy is defined as the deviation of a measure from the nominal value, and resolution is considered as the magnitude of measurable geometric features. As a consequence, accuracy and resolution of photogrammetric data heavily depend on the collection and processing strategy. To ensure the most accurate results, precise planning of acquisition and processing is crucial, whilst, most studies on archaeological stone tools present the results of analyses with little detail on the photogrammetric setup, data acquisition, and processing strategies.

The objective of this paper, structured according to rigorous “preproducibility” criteria (*sensu* [27]), is to draw attention to current protocols for 3D photogrammetric data acquisition and respond to the call made by Magnani and colleagues [9] to further discuss the limitations of current mainstream applications. It further aims to stimulate the debate towards achieving accurate results, which are essential when using this technique for analysis that goes beyond the subcentimetric detail of conventional applications. This paper serves not only as a proof of concept but also aims to suggest one of the potential solutions for improving the resolution and repeatability of experimental ground stone analysis. It also includes the characterization and quantification of surface depletion of two pairs of replicative GSTs (active and passive tools) at different stages of vegetable resource transformation, evaluating morphological changes in the tool’s entire geometry. This initial and promising test offers valuable insights into the tribological mechanisms that affect tools with diverse petrographic compositions [1]. By expanding the dataset, our results can serve as a proxy for evaluating archaeological evidence. In archaeological contexts one can only access a single stage of use (the last before its abandonment), hence lacking information about the other stages that occur throughout the stone tools’ biography. During the experimental documentation of the tribological mechanism, various steps are reproduced creating the conditions for meaningful comparisons to be made with archaeological items. The sequential replicative experiments, documented according to our methodological approach, is leading to the development of depletion models for understanding both the modification of the entire geometry and of the used surfaces texture. Therefore, our approach will enable a more informed approximation of the stages of use represented on the surfaces of archaeological stone tools, supporting their functional biography assessment.

### The contextualization of the experimental strategy

To investigate the cumulative development of use-wear on GST surfaces and measure their surface depletion, we built a reference collection for wear patterns that occurred during the processing of different plant organs (detailed in [23]). Our reference collection was constructed based on the GSTs retrieved in cultural level III of Brînzeni I, a cave located in the Edinet region, N-W Moldova [28]. The primary purpose of the site-specific reference collection was to understand the tribological mechanisms occurring during tool use, by investigating the cumulative development of use-wear on GST surfaces to establish proxies for comparing use traces and associated residues [23,29]. With this purpose, we set up a controlled sequential experiment, documented with a detailed protocol and standardised analytical strategy. To create the collection, riverine slabs and pebbles were selected, petrographically characterised, documented in their natural state, and finally used. Various issues related to the use of GSTs in processing aerial and underground plant organs were addressed. The experimental design included the selection of lithic resources based on archaeological evidence, ensuring their compatibility with the Brînzeni I GSTs in terms of morphometric features such as use-surface geometry, size, shape, weight, as well as petrographic characteristics [23].

The experimental stones replicative use, was structured according to sequential trials, considering repeated cycles of use lasting 30 minutes each, and labelled as “T”: T_0_ (unused), T_1_ (30 minutes of processing), T_2_ (60 minutes of processing), T_3_ (90 minutes of processing), and T_4_ (120 minutes of processing). During each experimental cycle, a pair of stones was used as passive and active tools to pound, crush, thresh, smash, and grind a single vegetal resource [23,29] selected among the one compatible with the biome of MIS 3, 60–25 ka (Marine Isotopic Stage 3) and according to the Brînzeni I pollen list [15,28,30–32].

**Fig 1:**
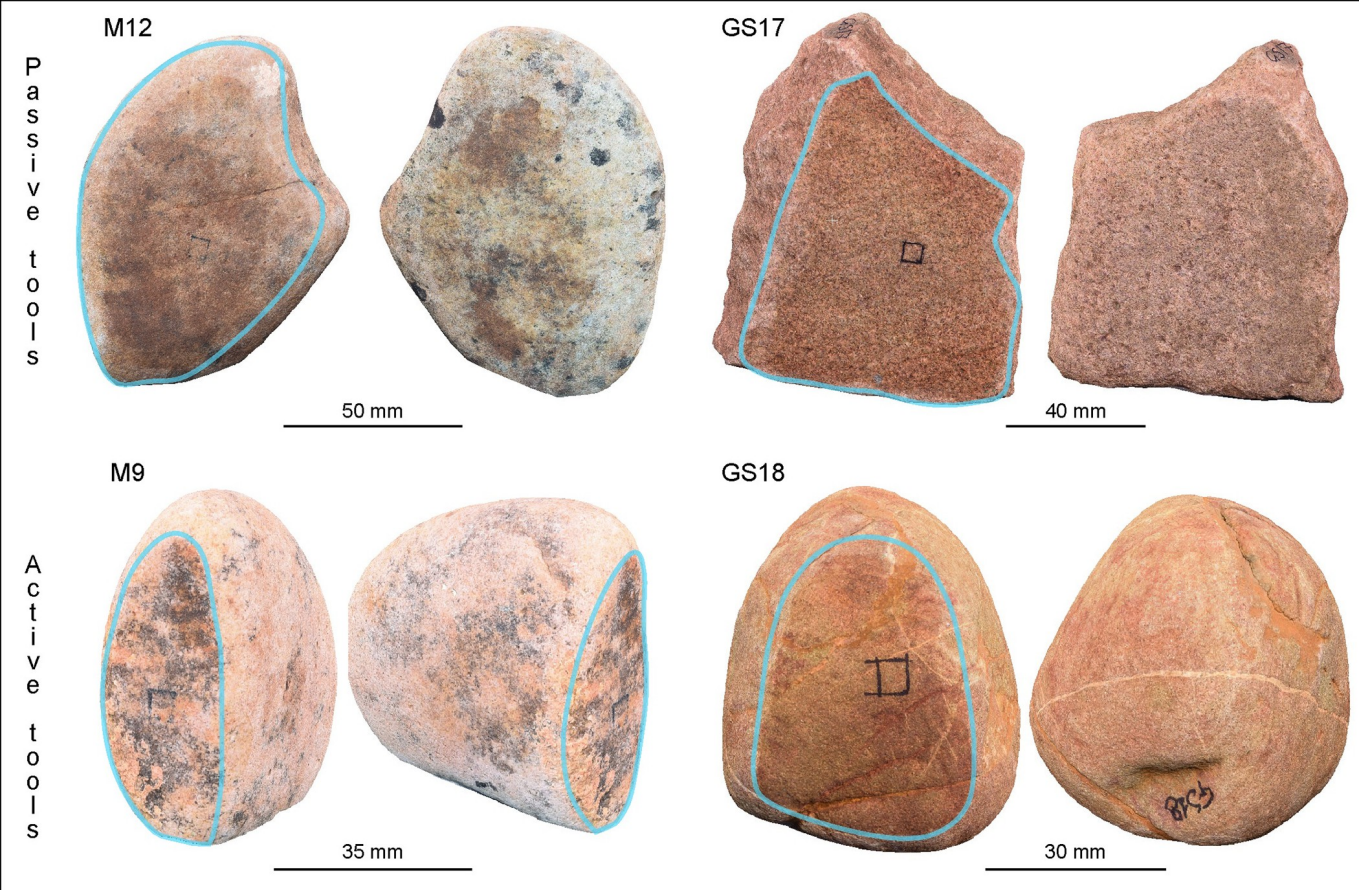
The 3D models of the GSTs involved in the experimental replica. The blue circle delineates the designated working surface of each tool.

For these pivotal assessment tests, we have included four GSTs replicas for analysis: M9, M12, GS17, GS18 (Fig 1). M9 and M12, are two quartz-arenite pebbles collected from the Racovăț River, flowing below the Brînzeni I ridge. The raw materials composing the pebbles are characterised by their high compactness and low porosity (<5%). Additionally, GS18 and GS17, consisting of litharenite and sandstone respectively, were collected from the Fiora River in Manciano, Italy, where sandstone of Miocene formation outcrops. These stones are primarily composed of quartz grains embedded in abundant carbonate cement, which makes them relatively softer compared to the Moldovan tool pairs. The four stone tools and their respective designated use surfaces exhibit distinct shapes based on their intended tasks, as indicated in Table 1.

**Table 1:** GSTs morphometric information. The weight, dimension, overall tool shape and selected use surface description are reported.

Stone	Weight (in g)	Dimension (in mm)	Tool overall shape	Use surface shape
GS17 (passive tool)	591.7	107.36×84.09×29.97	Flat trapezoidal prismatic shape	Flat profile
GS18 (active tool)	262.8	58.96×42.27×43.17	Spherical shape	Convex profile
M12 (passive tool)	427.5	100.38×80.57×29.5	Irregular shape	Concave profile
M9 (active tool)	215.9	62.73×29.81×57.67	Ovoidal shape (flattened)	Flat surface

M9 and M12 were used for 30 minutes (T_1_) while GS17 and GS18 were employed for a longer duration, a total of 2 hours (T_4_).

In this project multiscale data collection was performed documenting surface modifications at increasing levels of resolution and magnification, ranging from macro to sub-micro scales. The analytical strategy involves the recurrent recording of features before and after each replicative cycle. In order to capture the geometry of stone objects, 3D models were generated from photographic datasets. To investigate surface texture at micro to sub-micro scales, molds (obtained with PVS) of the stone surface were taken and observed with different microscopes. Additionally, the microtopography of specific controlled areas was reconstructed in 3D and measured using a confocal profilometer (for detail see [23,29]). The generation of 3D models and molds provided a multiscale documentation that not only enabled comparisons at any stage of the experiment, but also offered the added value of producing a permanent tool usage biography. While molds can consistently and persistently capture the micro and sub-micro scales of specific areas of the tool surface texture at the unused stage (T_0_) and throughout all the experimental cycles (up to T_4_), the 3D model generated by means of close-range photogrammetry allowed for a comprehensive analysis of the artefact at macro scale. This analysis reveals changes not only in localised areas of the surface (as microscopic studies do) but also in the entire geometry of the tool, which is the focus of this article. The multi-scale approach in its complex structure allowed for the characterisation and quantification of surface depletion, as well as modification/development of wear and preexisting natural features such as cracks and fractures, even on the unused stone faces.

Among the various techniques employed in the study, we are here reporting on the photogrammetric acquisitions, 3D elaborations and analysis. This, as the initial step of a multiscale recording and analytical strategy for wear formation and development, provides the extent and location of the surface contact areas as well as the macroscale quantification of surface depletion, that is further analysed and quantified by means of other techniques as microscopies, SEM, and confocal profilometer.

Photogrammetric techniques based on Structure-from-Motion (SfM) and Multi-View Stereo reconstruction were applied to generate 3D models of both passive and active tools used for the mechanical transformation of plant organs. Wear formation and their extent were examined by recording the geometry of each GST before and after use, and in particular at T_0_ and T_1_ for M9 and M12 while at T_0_ and T_4_ for GS17 and GS18.

Tests were conducted using different setups for data acquisition and elaboration assessing the reliability and resolution of the models by comparing the results of these tests and identifying potential sources of bias for discussion. As previously stated, the acquisition and elaboration of 3D models serve as the initial step in our comprehensive multiscale data acquisition and analytical strategy. The aim was to identify changes in the overall geometry of the tools and macroscopically determine the location and the extent of the contact area. Subsequently, these zones can be subjected to further detailed analysis using quantitative and qualitative methods.

Photogrammetry was preferred over 3D scanning due to its cost-effectiveness, accessibility, as well as its portability for fieldwork in archaeological and museum studies. Furthermore, its flexibility allows us to adjust the resolution of the output 3D model according to the needs of our research question, a parameter that can be difficult to anticipate at the outset of a study. The morphological changes of the tools are highly dependent on artefacts’ lithic characteristics, the medium processed, and length of use making it difficult to predetermine the needed resolution (even though one can expect morphological changes in the range of the tenth of a millimetre) for capturing these use-related features. Photogrammetry, by enabling us to adjust the resolution during the study as needed, proved to be crucial for our research. Additionally, the future steps of the study will entail the comparison of the 3D experimental data with the 3D data from the Brînzeni I archaeological GSTs. This will require securing comparable data using photogrammetric technique. To accomplish this, and in consideration of the intention to expand the dataset to include a larger number of archaeological sites, visits to foreign museums and archaeological sites will be necessary, highlighting the need for flexible and easily transportable equipment.

**Fig 2:**
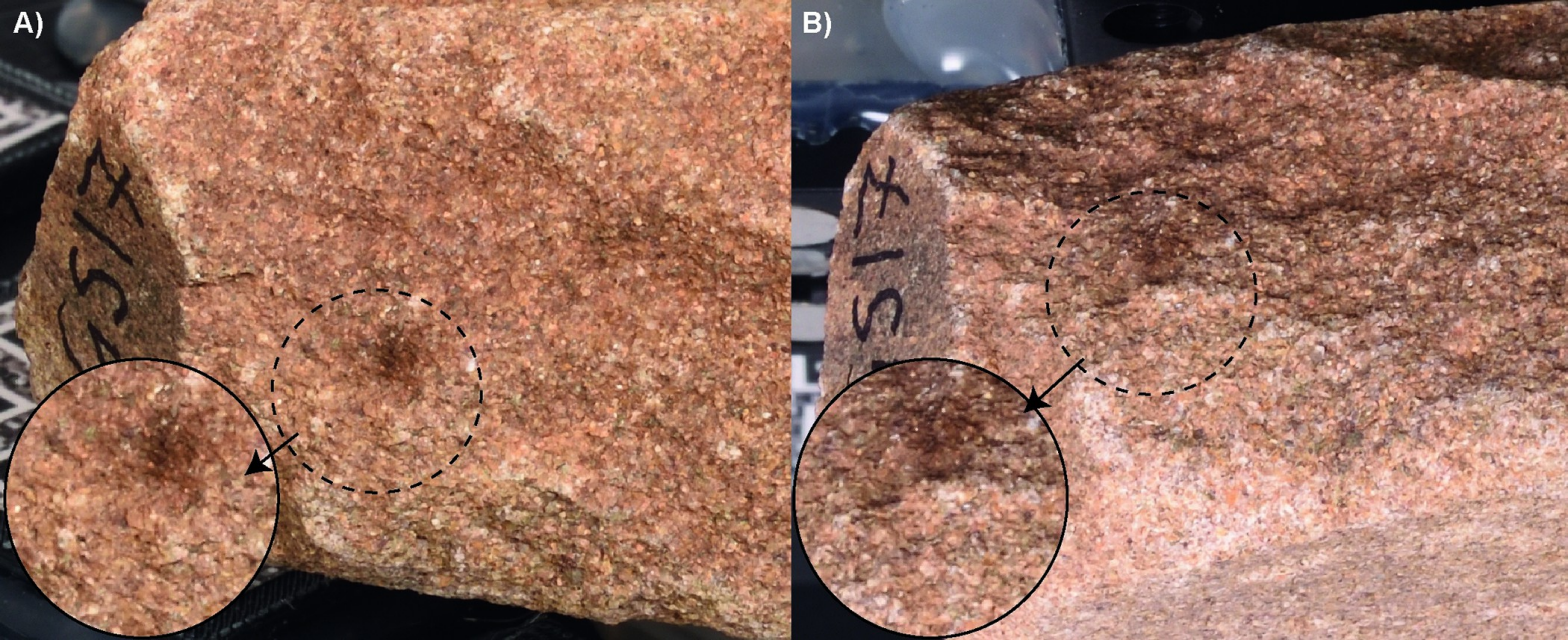
Example of the dependency of the local texture on the shades casted by directional lighting when moving/flipping over the stone on the acquisition space. The same area of the stone rim is depicted A) with the use surface facing up and B) with the use surface leaning on the turntable.

It was evident from the outset of preliminary tests that the photogrammetric technique may be prone to typical errors and limitations that can have a significant impact on the analysis. Among them, the scale factor error is an issue already raised by different scholars (e.g., [13,33]). Photogrammetric models are typically scaled by including at least one scale bar into the scene so that its known length can be transferred to the 3D model. The process requires the collimation of markers on the scale bar during data processing, which in the case of manual measurement can introduce significant human errors of several micrometres. Another issue that arose when comparing the models of the tools before and after usage was that they were not referenced and oriented according to the same spatial coordinates. Since the surface of these objects is highly uneven with some porosity, moving them in the acquisition space can change shadow direction modifying the texture appearance and thus negatively influencing the photogrammetric process (Fig 2) [34]. To overcome these challenges, various strategies were explored during data acquisition and processing.

## Materials and methods

This paper focused on assessment tests conducted on four different experimental GSTs used in pairs to process the achenes of *Rumex crispus*. This resource was chosen for its small, hard, and rounded pericarp that are subject to repeated direct contact between the two lithic surfaces during grinding, as a result of achenes scattering onto the passive tool. This determines a fast and intense stone surface depletion. The two pairs of tools were used for a different length of time: M9 and M12 were used for a duration of 30 minutes (T_1_) resulting in a limited surface depletion. Consequently, GS17 and GS18 were employed for a longer duration, a total of 2 hours (T_4_) to ensure an appreciable reduction of the surface.

To examine wear formation and extent we first reconstructed in 3D each tool at the T_0_ stage using photogrammetry. The obtained 3D models were later compared with those of the T_1_ stage for M9 and M12, and to the T_4_ stage for GS17 and GS18.

### Settings

**Fig 3:**
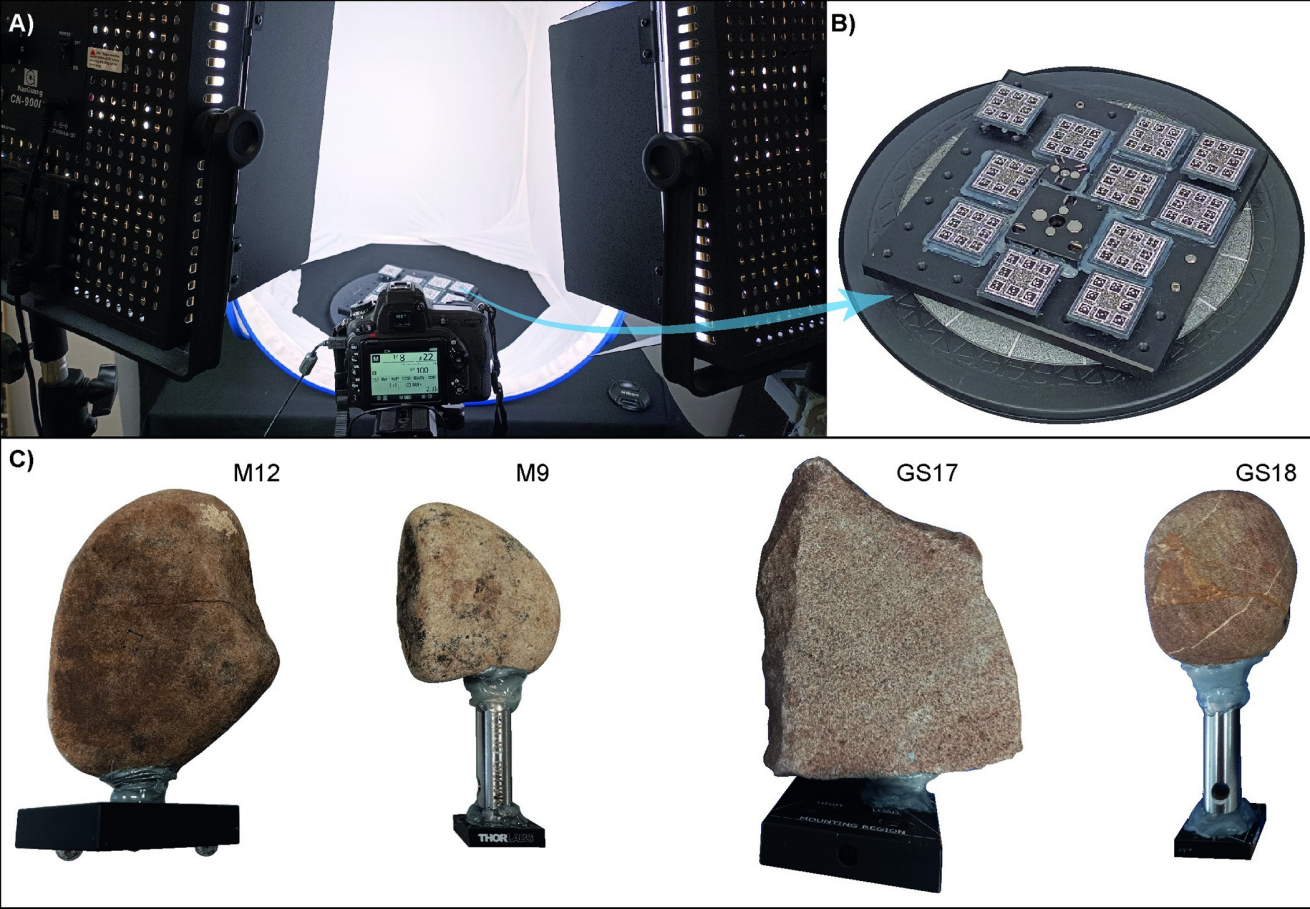
The photogrammetric acquisition setup in the FBK lab. A) The light box, two symmetrically placed LED light panels and the Nikon D750 camera in the middle with camera settings displayed. B) The turntable, equipped with fixed markers and with the bottom plates of the kinematics bases. C) The GSTs involved in the study supplied with the top plates of the kinematics bases.

The set-up of the photogrammetric acquisition remained consistent across all tests (Fig 3). The GSTs were individually placed on a rotating table with fixed markers at various heights (see Fig 3B). To ensure even lighting of the sample, a light box and two symmetrically placed LED light panels were employed. A Nikon D750 camera equipped with an AF-S Micro NIKKOR 60 mm lens was used to acquire the photographs (Fig 3A). The same camera settings were applied for all acquisitions, using manual settings. To prevent involuntary focus changes, the focus ring was locked with hot melt glue. To minimise noise, the ISO was set to 100 native value. Prior to acquisition, different lens apertures were tested to maximise the depth of field without losing image sharpness (due to diffraction), and a F-stop of 22 was ultimately selected. Due to the low ISO and high F-stop, a slow shutter speed of approximately one-eighth of a second was chosen. The setup and camera parameters were calibrated to always achieve a ground sampling distance (GSD) between 0.0413 and 0.0548 mm. To reduce the risk of motion blur, both a remote shutter and the function to raise and lock the mirror up before shooting were employed too. For the same reason, the camera was placed on a tripod standing in front of the table and in between the two lights. The pictures were acquired in NEF raw format (.nef).

### Acquisition strategies

Two different acquisition strategies were tested to acquire the photogrammetric data.

The first setup was *ad hoc* constructed for the case-study. Two distinct metrological kinematic bases, manufactured by ThorLabs, were used: the KB1X1 and KB2X2 (Fig 3B and 3C). Each base comprises two plates, one with three spherical earth magnets and the other with three V-grooves, which allow for the top plate to be positioned on the base plate on the same location and orientation with high precision and repeatability (KB1X1: minimum error: 0.58 μrad, maximum error: 26.72 μrad; KB2X2: minimum error: 3.60 μrad, maximum error: 30.96 μrad) [35]. The base KB1X1 was employed for the active stones while the KB2X2 for the passives. Each stone was perforated to a depth of approximately 0.5 cm, and thread-to-thread adapters manufactured by Edmund Optics for optomechanical applications, were inserted and fixed in place with a two-component epoxy metal glue. The opposite ends of each adapter were attached to the top plates of the kinematic bases and secured with hot melt glue (Fig 3C), while the bottom plates were fixed to the turntable (Fig 3B). This guarantees that the stones can be placed and removed from the rotating plate each time a replicative experiment is performed, and then repositioned on it with high precision, ensuring always the same position and orientation of the stone (within the uncertainty defined by the manufacturer).

**Fig 4:**
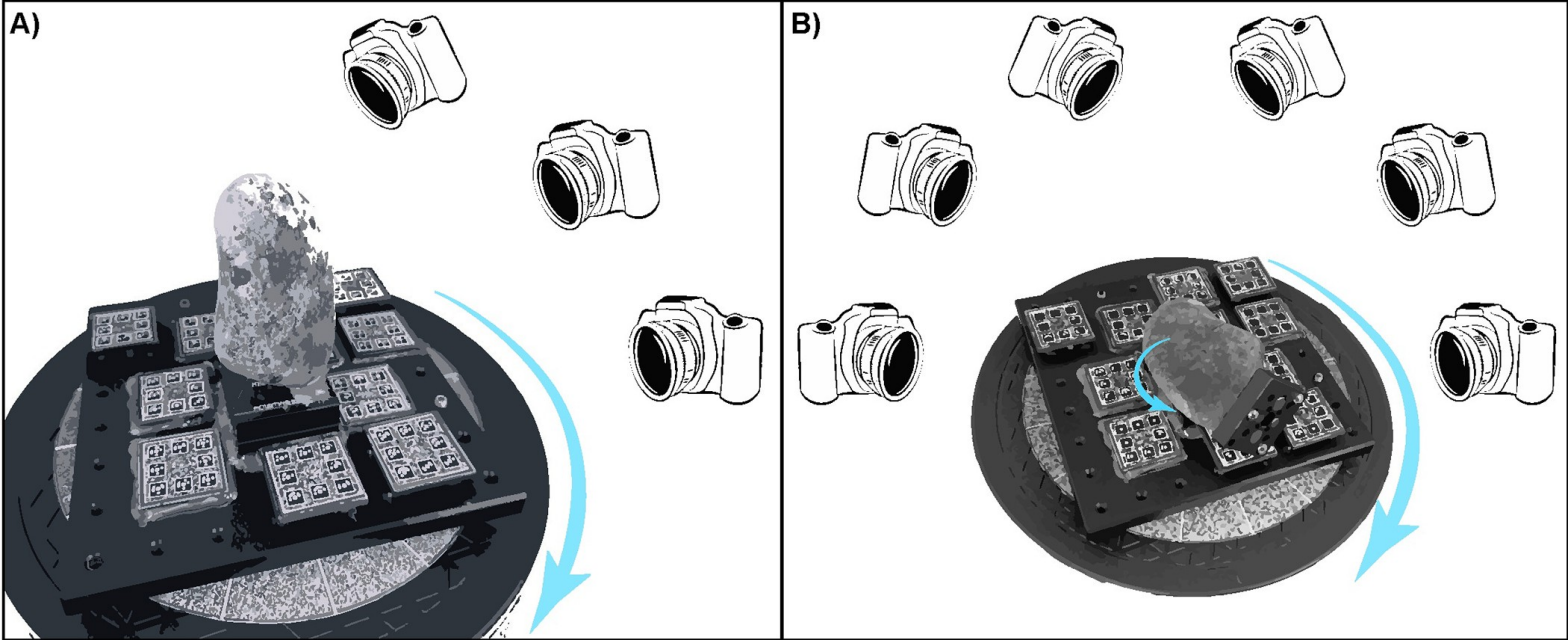
The two different acquisition setups. A) The *ad hoc* setup: the items are fixed on the turntable employing the kinematic bases and pictures acquired adjusting the position of the camera at three different heights. B) The second setup: the items are placed horizontally on the rotating plate and pictures captured adjusting the position of the camera at three different heights. Subsequently, the object is flipped over to capture pictures of its other side and again pictures are captured adjusting the position of the camera at three different heights.

Pictures were captured by rotating the table at intervals of 10-degrees (or less in proximity to the narrow edges of the stones) and adjusting the position and tilt of the camera towards the object at three different heights (Fig 4A). Additional photographs were taken of complex, top and/or narrow areas of the items resulting in an average of 100–120 images.

The acquisition of each GSTs was repeated 3 times for each T of the experiment to ensure repeatability and to estimate the uncertainty of the acquisition method (T_0_ acquisition 1, 2, 3; T_1_/T_4_ acquisition 5, 6, 7).

In the second setup, the objects were placed horizontally on a rotating plate instead of being fixed. We followed the acquisition strategy outlined in the available literature for digitising stone tools (Fig 4B) (e.g., [9,13–15,17,24,33]). In this setup pictures were captured by rotating the plate at 15-degree intervals and adjusting the position and tilt of the camera towards the object at three different heights. Subsequently, the object was flipped over to capture pictures of its other side, resulting in two sets of around 75–95 photographs (for a total of ca. 150–190 pictures) for each experimental T (T_0_ acquisition 4; T_1_/T_4_ acquisition 8).

The difference in the rotation degree angle between the commonly used method in the literature and our setup can be attributed to the specific approach we adopted. In the traditional method, pictures are acquired at three different heights and then the object is turned, resulting in six subsets of pictures that adequately cover the object. This allows for a larger rotation degree angle of the turntable, typically around 15 or even 20 degrees. However, in our setup, we made a deliberate choice to avoid turning the object, resulting in three subsets of pictures. To ensure comprehensive coverage of the entire object and minimize any potential gaps in the data we increased the number of pictures taken by rotating the turntable every 10 degrees or even less. This adjustment was necessary to maintain the accuracy and completeness of our data acquisition process.

In order to calibrate the camera parameters, consistently orient the models of the stones at different T to a shared spatial coordinate system, and ensure a resolute and consistent model scaling, a separate calibration project was carried out, which involved acquiring the setup without any samples. For scaling purposes, a low thermal expansion scale bar (Brunson 803-MCL Length Reference Kit) was fixed to the rotating plate. This tool, made of Invar alloy (FeNi36), provides a thermally stable reference length of 224.9998 mm [36]. During this process, images of the turntable with the markers were captured at 20-degree intervals. The camera was adjusted to obtain images at three different heights, and was also rotated horizontally to the left and right (i.e., both portrait and landscape orientation), following standard protocols of a self-calibration imaging network [37]. Although camera calibration can be part of the SfM process and is regularly used in many 3D digitisation projects carried out using photogrammetry, an *ad hoc* project for camera calibration ensures that the most suited camera network is realised. This is in particular important for high accuracy projects where the imaging network is not optimised for calibration but for the 3D digitisation of the object of interest.

### Data processing

The digital raw pictures were converted from.nef to.jpg format using NxStudio, and then processed using Agisoft Metashape software to generate the 3D models.

The calibration file for the camera settings and markers coordinate was imported into each individual project to ensure accurate and consistent calibration before processing the photographs of the artefacts. The stone tool pictures were imported in new projects into the software which oriented the images and automatically detected the markers present on the rotating plate. The markers coordinate system was imported to ensure consistent spatial orientation and object scaling across all projects. Finally, the dense cloud and mesh were generated and texturized to produce the final 3D model, which was exported in.obj format. For the acquisitions performed according to the second setup (strategy typically available in literature) some more steps were needed, including masking one side of the object for the correct orientation of the pictures, a time-consuming procedure on a large dataset, and in some case (GS17 at T_0_ acquisition 4) the images of the two side of the stone had to be oriented in separated chunks that were subsequently merged into a single point cloud. Nevertheless, all 3D models are missing the side of the stone where the pin is attached, which should not influence the analysis since it is not involved in the resource processing.

Summarising, in the end of the data processing for each of the four GSTs involved we obtained:

- 4 models of the stones unused (T_0_): 3 acquired with the proposed setup (named acquisition 1, 2, 3), and 1 acquired with a setup commonly used in 3D digitisation projects (named acquisition 4);
- 4 models of the stone tools after *Rumex crispus* achenes processing (T_1_ in case of M9-M12 and T_4_ for GS17-GS18): 3 acquired with the *ad hoc* setup (named acquisition 5, 6, 7), and 1 acquired with a setup commonly used in 3D digitisation projects (named acquisition 8).

Each.obj file was imported into the open-source software CloudCompare to be analysed.

The Cloud-to-Mesh Distance function was employed to compare all GSTs at T_0_ with the T_1_/T_4_ meshes. The Cloud-to-Mesh Distance computes the gap between two entities, a cloud and a mesh, or two meshes as in this case, calculating the distance of the vertices of the compared mesh to the triangles of the reference mesh. The analysis is presented in the next paragraph and displayed in false colour images indicating the distance between the reference item (T_0_ models) and the model of the tool after its use, which served as the comparison. The colour gradient in the images ranges from red to blue and includes intermediate tones such as orange, yellow, green, and blue. The values in the red, orange, and bright yellow regions indicate a positive distance, with a maximum of 100 μm represented in red. The yellow and acid green tones indicate no distance, while the shades of green, turquoise, and blue represent a negative distance, with a maximum of -400 μm depicted in dark blue. Any distance exceeding the reference range of 100 μm to -400 μm is presented in a uniform shade of grey.

For each stone at T_0_, the photogrammetric survey was repeated three times to investigate the repeatability of the method, and verify the repositioning error of the kinematic bases (acquisition 1, 2, and 3 compared among them). Subsequently, the three models of the same sample at T_1_/T_4_ were also compared to enhance the statistic (acquisition 5, 6, and 7 compared among them). Finally, the T_0_ models were all compared with the T_1_/T_4_ models to quantify surface depletion during use.

To overcome the repositioning error of the base, a subsequent attempt was performed involving the Iterative Closest Point (ICP) function of CloudCompare. This is a registration algorithm that iteratively aligns two entities. In this case the T_0_ acquisitions were used as the reference files and the T_1_/T_4_ models translated and rotated to match the reference. The ICP algorithm attempts the alignment of the two entities until the difference in error (RMS) between two iterations falls below a predefined threshold that was set to 95% (5% difference was estimated due to use-related morphological change). Furthermore, to improve computational efficiency, the Random Sampling Limit was set to 500000, which subsampled the data cloud at each iteration, and the Enable Farthest Point Removal option was enabled to discard possible points that are too distant from the model cloud and thus may represent potential outliers.

Again, the models T_0_ acquisitions 1, 2, and 3 of each GSTs were compared with the models T_1_/T_4_ acquisitions 5, 6, and 7.

Also, the models T_0_ acquisition 4 were aligned and compared with the models T_1_/T_4_ acquisition 8.

During the processing of raw resources using GSTs, the entire stone undergoes transformation, however, the surfaces directly involved in the activity experience the most significant wear. Therefore, attempting to find an orientation that minimises the difference between the two models acquired before and after the tool’s use can lead to errors. Consequently, an additional test was conducted to align the T_0_ and T_1_/T_4_ models using only the part of the stones that is not directly involved in the grounding/pounding task. The 3D models were cut, removing the active surface of each tool model, and the remaining parts were again finely registered between each other using the ICP algorithm. The transformation matrices occurring during the co-registration of T_0_ and T_1_/T_4_ half models were then imported into the T_1_/T_4_ entire models. Then the models T_0_ acquisitions 1, 2, and 3 of each GSTs were compared again with the transformed models T_1_/T_4_ acquisitions 5, 6, and 7. The same procedure was also applied for the comparison of T_0_ acquisition 4 with T_1_/T_4_ acquisition 8.

## Results

**Fig 5:**
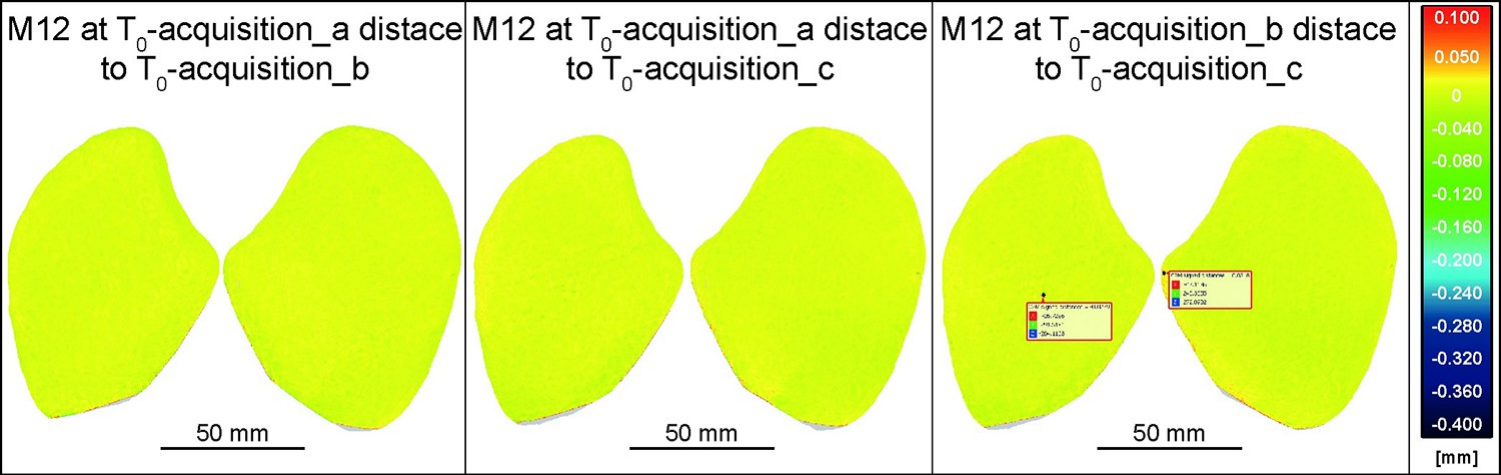
M12 at T_0_ acquisition a, b, c compared among them. This test was necessary to verify the acquisition errors unrelated to the kinematic base repositioning variances.

The analysis began by assessing the repeatability of the implemented photogrammetric process. In order to verify the repeatability of the acquisition and the putative errors, one more test was performed on tool M12 at T_0_ (natural surface, not used). The stone was placed on the turntable and fixed with the kinematic base, and consequently acquired three successive times, without removing it from the turntable to avoid introducing the repositioning error by the operator and/or the kinematic base. The three obtained meshes were named acquisition a, b, and c, and they were compared to one another. Since there is no surface depletion among the models acquired at the same T, any observed variation should primarily result from acquisition errors. The results showed a high level of overlap with only a few singular spots showing an absolute distance of up to 28 μm due to acquisition noise (Fig 5).

**Fig 6:**
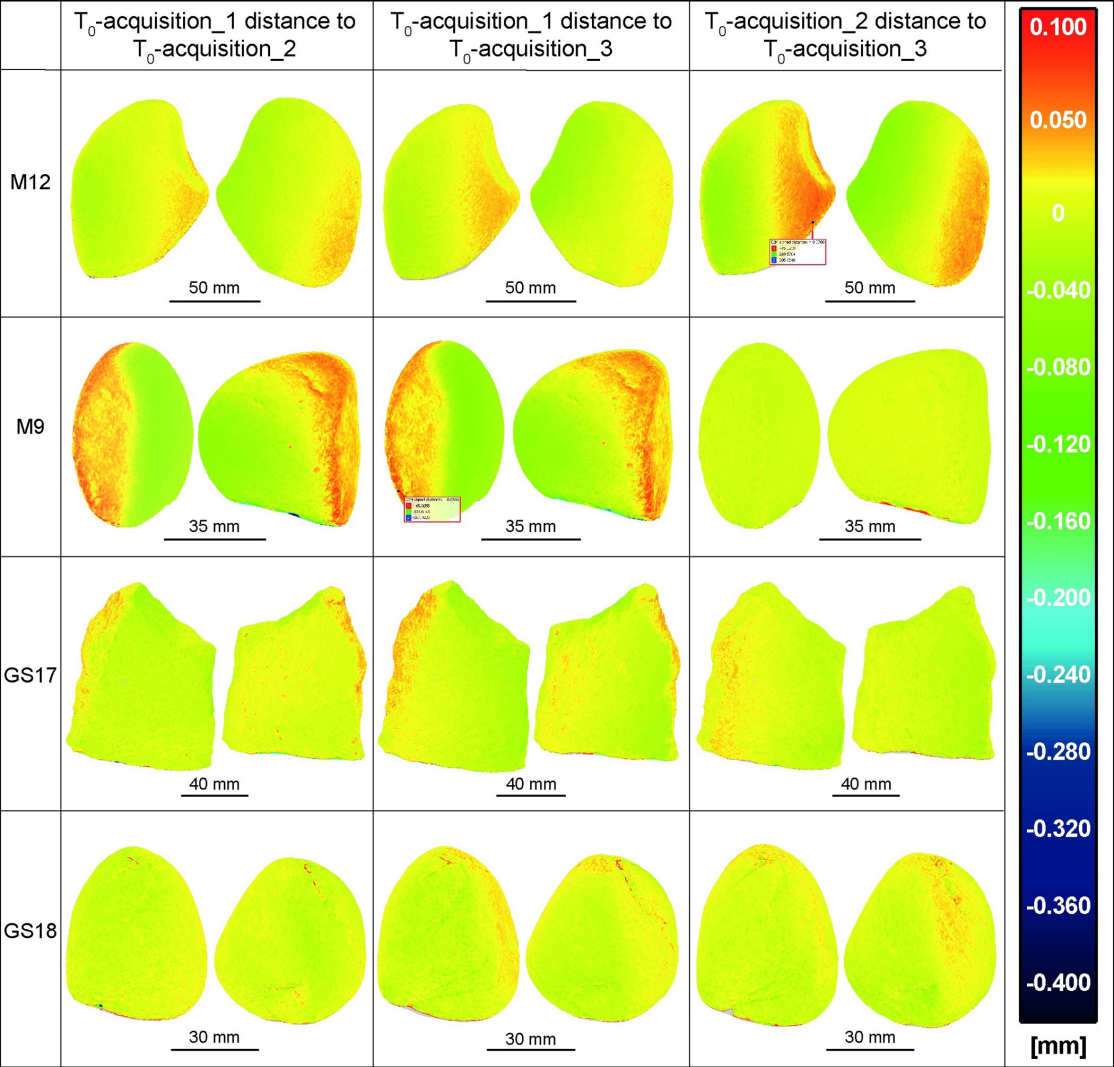
All the involved GSTs at T_0_ acquisitions 1, 2, and 3 compared among them. The analysis showed a high degree of overlap between the compared models, with an average absolute distance of approximately 40 μm. Only in some cases, such as when comparing M9 between acquisition 1 and 3, as well as 1 and 2, and M12 between acquisition 2 and 3, a few limited areas, such as the edge of the surface, presents a maximum distance of 80 μm.

**Fig 7:**
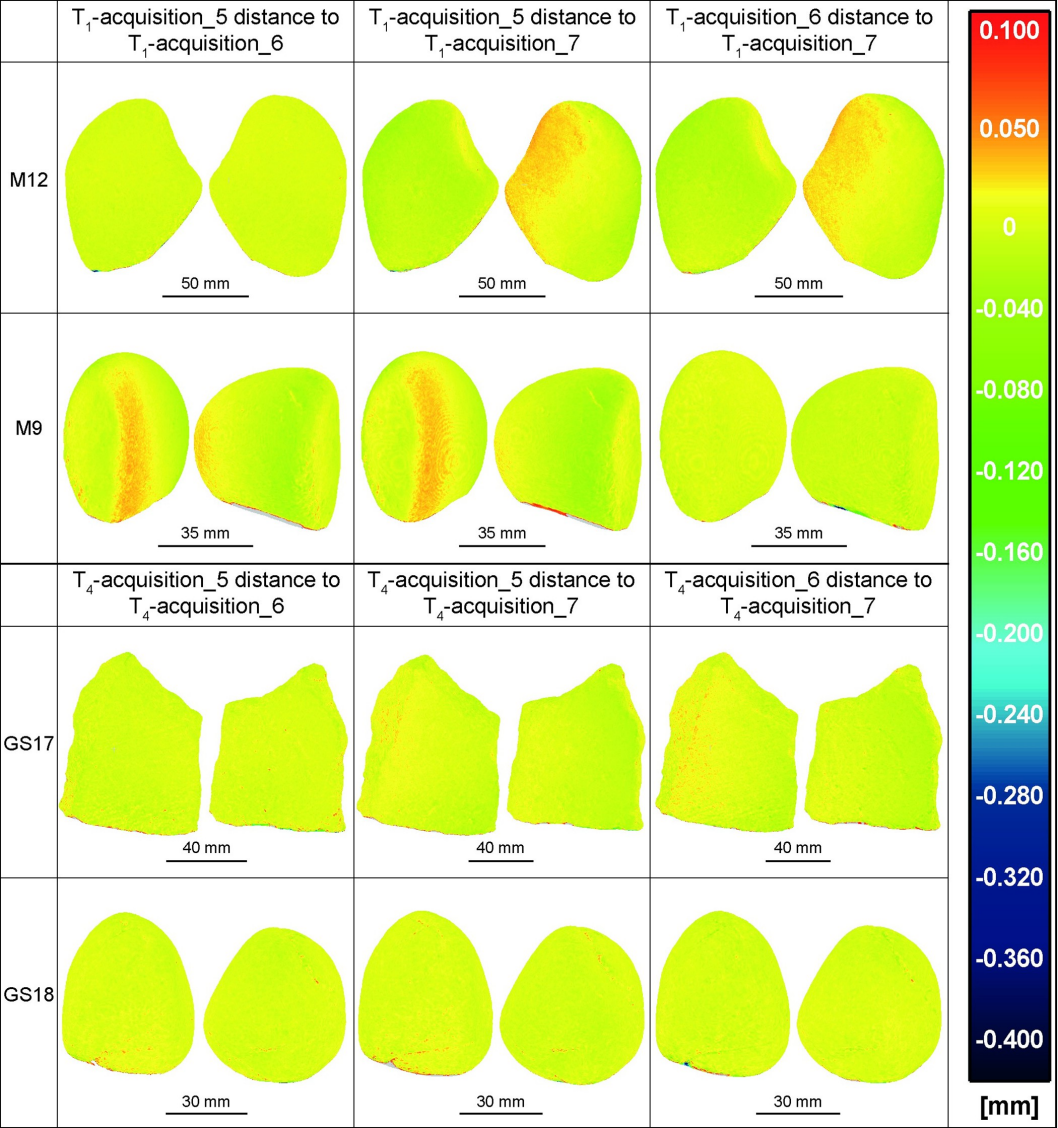
All the involved GSTs at T_1_/T_4_ acquisitions 5, 6 and 7 compared among them. The results indicate a high degree of overlap between the compared models, suggesting a significant level of repeatability in the repositioning of the items achieved through the use of kinematic bases.

Further, to verify the kinematic bases inaccuracy in repositioning a comparison was made among all GSTs models at T_0_ acquisition 1, 2 and 3 (Fig 6) and separately among the T_1_ and T_4_ acquisition 5, 6, and 7 (Fig 7). The outcomes indicated a substantial degree of overlapping among the compared models, with an average absolute distance of approximately 40 μm (represented by green and orange areas) and a maximum of 80 μm (limited to red and darker green areas), such as in the case of M9 and M12 at T_0_ (Fig 6).

**Fig 8:**
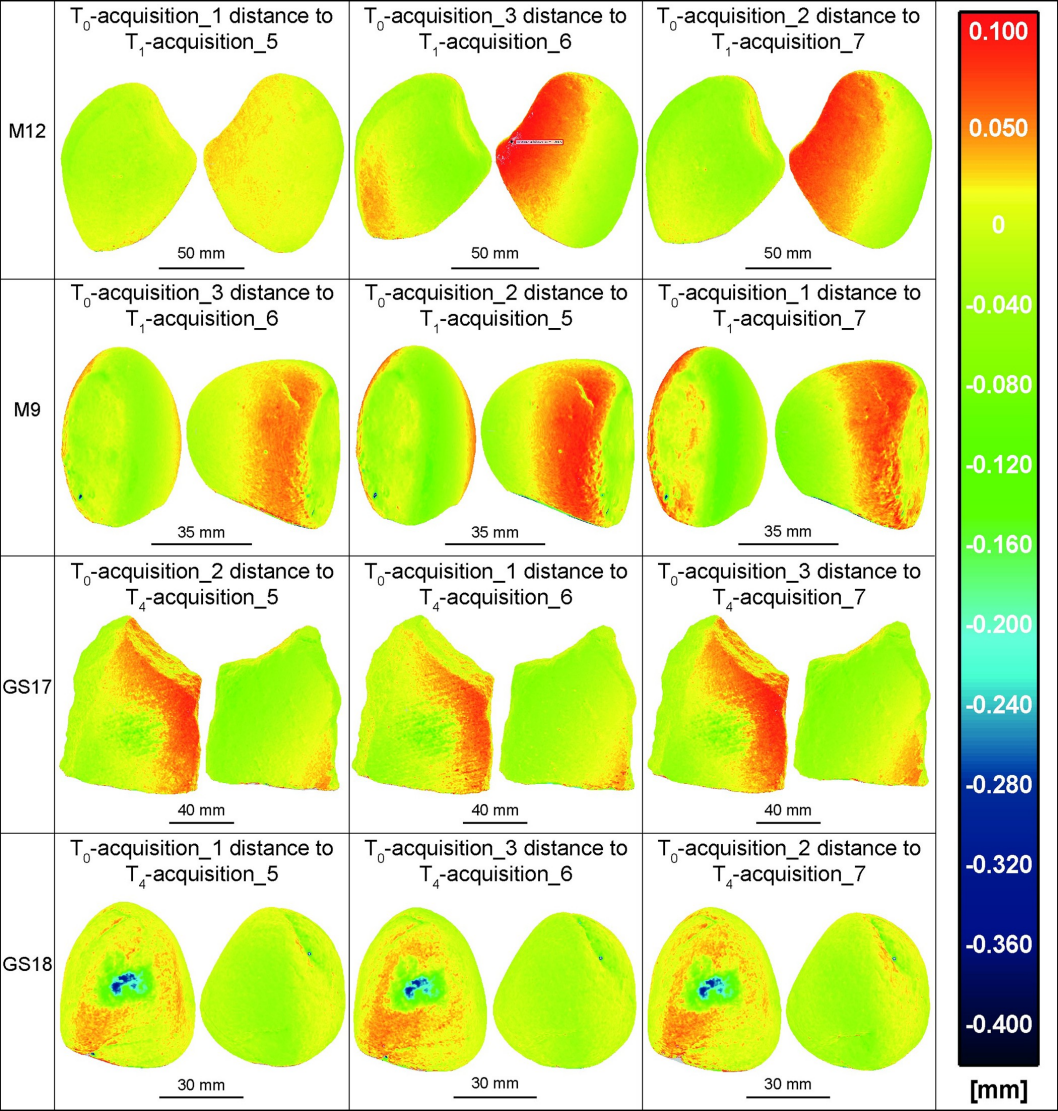
The acquisitions 1, 2, and 3 of all GSTs at T_0_ compared with the acquisitions 5, 6, and 7 of the same GSTs at T_1_/T_4_. The false colour images depict the distance between the mesh of the unused items and the mesh of the tools after use, aiming to highlight distinct wear patterns. The analysis revealed that wear patterns were particularly noticeable in the central region of the working surface of the tools GS17 and GS18, which are made of a softer rock type and were used for a duration of 2 hours.

**Fig 9:**
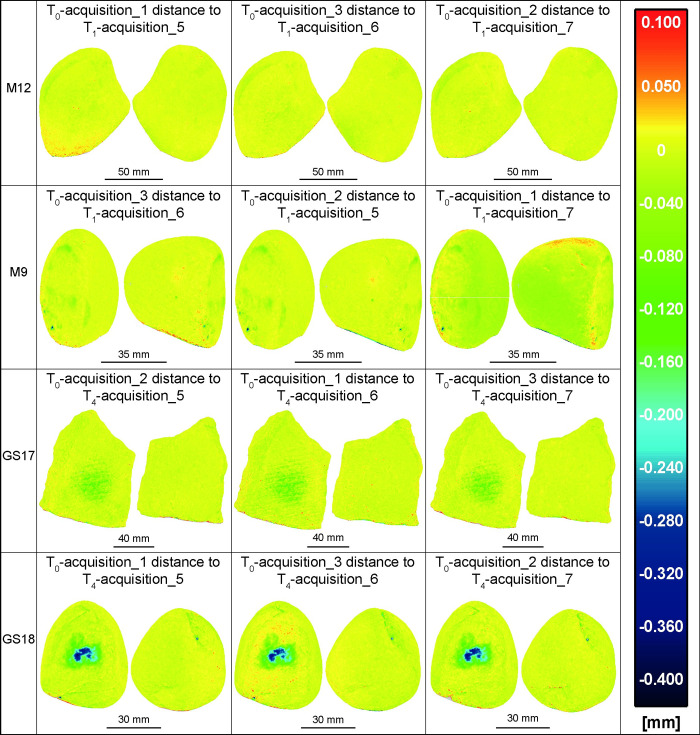
The acquisitions 1, 2, and 3 of all GSTs at T_0_ compared with the acquisitions 5, 6, and 7 of the same GSTs at T_1_/T_4_ applying the ICP algorithm. False-colour images display the distance between the mesh of the unused items and the mesh of the tools after use. The application of the ICP fine registration algorithm further enhanced the visibility of wear patterns, including those on M12 and M9, which had less intense depletion due to their shorter use periods.

**Fig 10:**
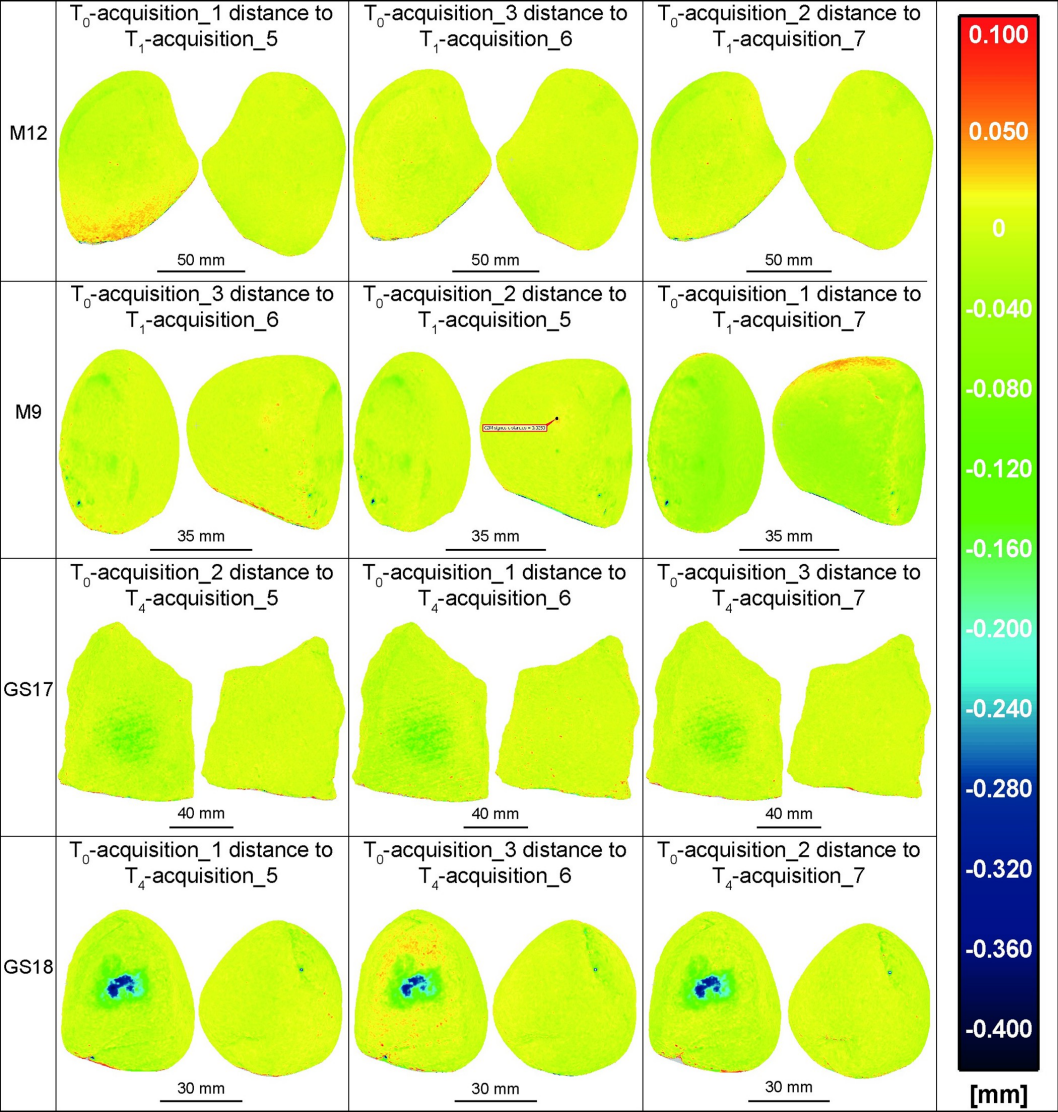
The acquisitions 1, 2, and 3 of all GSTs at T_0_ compared with the acquisitions 5, 6, and 7 of the same GSTs at T_1_/T_4_ applying the ICP algorithm on the unused part of the tools. The results do not show a significant difference compared to the previous test illustrated in Fig 9.

The use of stone tools leads to a gradual loss of material through subtractive adhesive, fatigue and abrasive wear mechanisms (e.g., [38]). Therefore, it is expected that the differences between T_0_ and T_1_/T_4_ should be zero or negative in the used surface. To estimate the surface depletion of each GSTs, we compared their T_0_ models with the models of the tools after their use in processing *Rumex crispus* (Figs 8–10; Table 2). The M12 rock type is particularly resistant to mechanical stress and the use time was limited to 30 minutes, therefore changes in the working surface are barely appreciable. Indeed, the distance between T_0_ and T_1_ models are up to -40 μm, primarily along the working surface’s edges and a superficial but widespread wear pattern is also apparent in the central region.

**Table 2:** Summary of the depletion observed for each GSTs. It includes information on the location of wear on the used surface and the maximum extent of the depletion when compared to the unused stone surface (T_0_).

Stone	Depletion assessment between T_0_ and T_1_/T_4_	Area interested by the depletion
M12	up to -40 μm	Primarily along the working surface’s edges and widespread wear pattern in the central region
M9	up to -70 μm	On convex central area
	up to -370 μm	Few battering marks
GS17	up to -170 μm	Covering a large central area of the used surface
GS18	up to -360 μm	Extending on the protruding area of the convex use surface

On the other hand, the active tool M9 at T_1_ displayed more pronounced wear patterns compared to M12, particularly in the convex and protruding areas of the use surface (green areas displaying a distance to T_0_ up to -70 μm). Additionally, few battering marks were also detected, presenting a maximum depth of -370 μm, represented in the false-colour analysis as dark blue spots.

As expected, the pair of GS17-GS18 tools, made of a softer stone type and employed for a longer period of 2 hours in achenes processing, presented a more intense wear pattern. GS17 at T_4_ displayed a large depletion area in the centre of the used surface, whose distance from T_0_ was up to -170 μm (dark green region). The active tool GS18 at T_4_ presented the most defined and intense wear pattern, extending on the protruding area of its convex use surface. The distance to T_0_ for this area reached up to -360 μm. Additionally, cracks were observed on the unused dorsal surface (the side held in the hand), which deepened after stone use, confirming that the entire tool underwent a morphological change while performing a transformative task.

Lastly, despite the cleaning process that each tool underwent after their use, all exhibited flour residues of *Rumex crispus* embedded in surface craters, depicted in the analysis as isolated red dots. The attribution of these positive distance spots to flour residues was confirmed by microscopic observation of the GSTs [23,29].

Comparing models based on the alignment provided by the kinematic bases has proven to be effective in highlighting and measuring wear patterns (Fig 8). However, the positioning inaccuracy, in particular the rotation of the kinematic bases may result in distances between models that exceed 100 μm in absolute value, even in areas unmodified by the replicative tool usage. In some cases, these errors can be greater than the mesh distance due to surface depletion. This was observed in the case of M12 T_0_ acquisition 3 when compared to T_1_ acquisition 6, where the repositioning error resulted in a distance of up to 103 μm in absolute value, which is greater than the -30, -40 μm variation observed on the working surface. Similar results were observed in the comparison between M9 T_0_ acquisition 2 and T_1_ acquisition 5. Otherwise, the distance caused by repositioning errors did not exceed 100 μm for GS17 and 50 μm for GS18, and did not overlap with the wear patterns observed.

Applying the ICP fine registration algorithm to the already well-aligned models further minimises the distance errors, improving the recognition of the actual distance due to wear mechanisms (Fig 9). The ICP algorithm translate and rotates an item compared to a reference item to minimise their metric differences. Since our aim was to highlight and measure these differences, we also tested co-registering only the unused half of the tools before analysing the whole objects (Fig 10). While methodologically this approach may seem more appropriate, the outcome did not show a significant difference compared to the previous approach. This lack of significant difference could be attributed to the ICP implementation in CloudCompare, which automatically excludes points that are considered outliers and are too distant from each other when the "Enable Farthest Point Removal" function is enabled.

**Fig 11:**
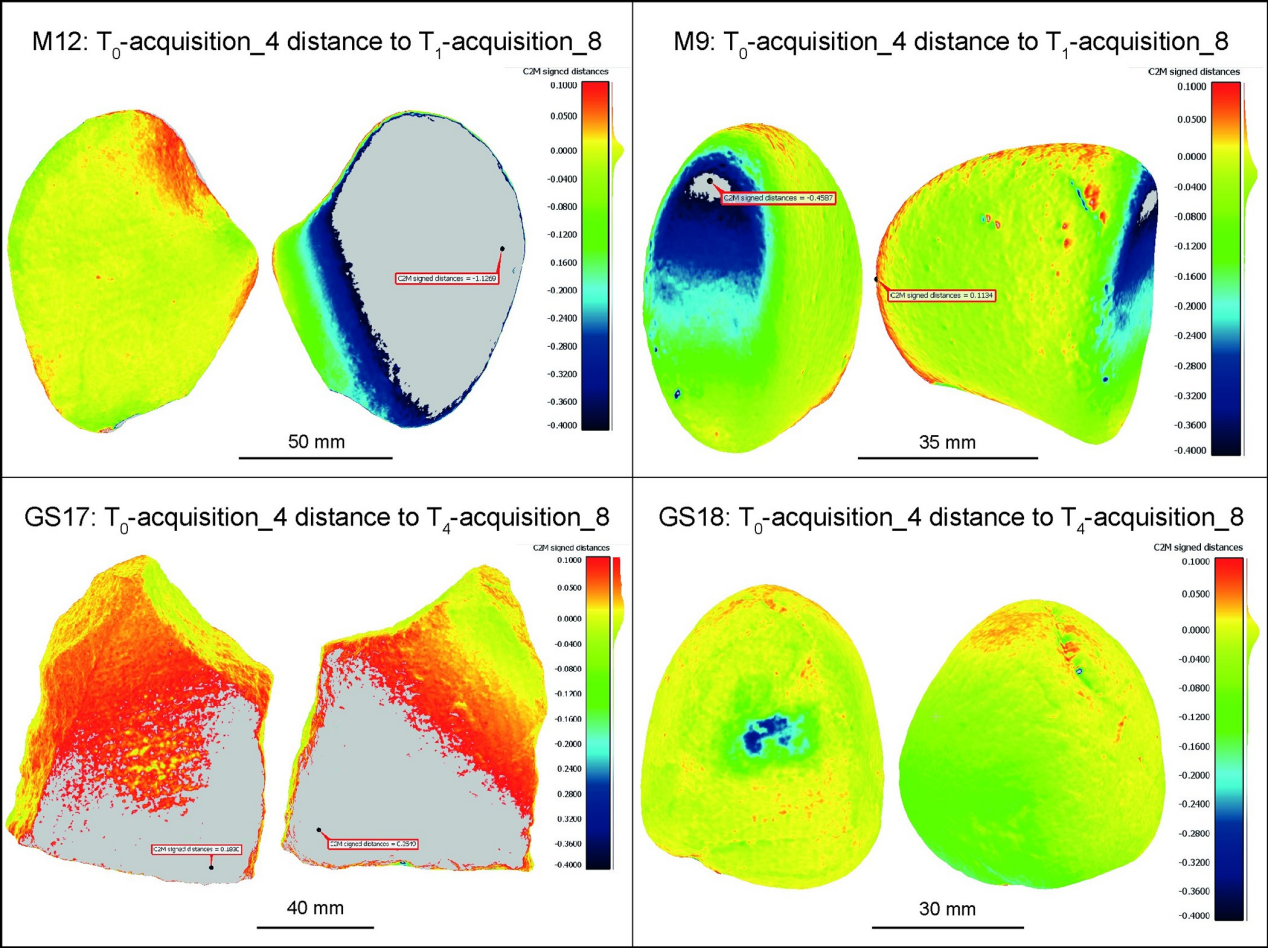
Acquisitions 4 of all GSTs at T_0_ compared with acquisitions 8 of the same GSTs at T_1_/T_4_. The data acquisition and elaboration were performed following literature guidelines. The comparison is performed using the ICP algorithm for the alignment and computing the mesh-to-mesh distance. In all items, except GS18, the distance exceeds the reference scale and in all cases the distance due to alignment overcomes the 100 μm in absolute value.

It should be noted that relying solely on the ICP algorithm for fine alignment without implying the kinematic base may not be sufficient to achieve the desired level of interpretation. Tests adhering to literature guidelines for data acquisition and elaboration, and considering rough manual positioning followed by ICP alignment refinement, resulted in distances exceeding 100 μm in areas unaffected by wear, reaching up to 2.5 mm in the case of GS17 or completely covering the wear patterns, as for M9 (Fig 11). Additionally, acquiring the two faces of the object as separate sets can add errors to the photogrammetric process of image orientation when trying to merge the two image sets. These errors are more likely to occur when the illumination is not properly diffused because the shadows casted by the micro topography of the stones depend on the relative position of the stone themselves and the light sources [34,39]. Indeed, the photogrammetric process in automated algorithms like SfM rely on the detection and matching of natural interest features of the object (e.g., circular blobs, corners) between the images. These features in well textured objects are: i) numerous and ii) discriminative (i.e., each point can be uniquely distinguished from another) making them ideal for a reliable photogrammetric process. The dependency of the local texture on the shades cast by directional lighting may be the reason for recurrent errors when orienting images of two sets taken after flipping over the stone on the turntable (Fig 2), as highlighted in previous study [40].

**Fig 12:**
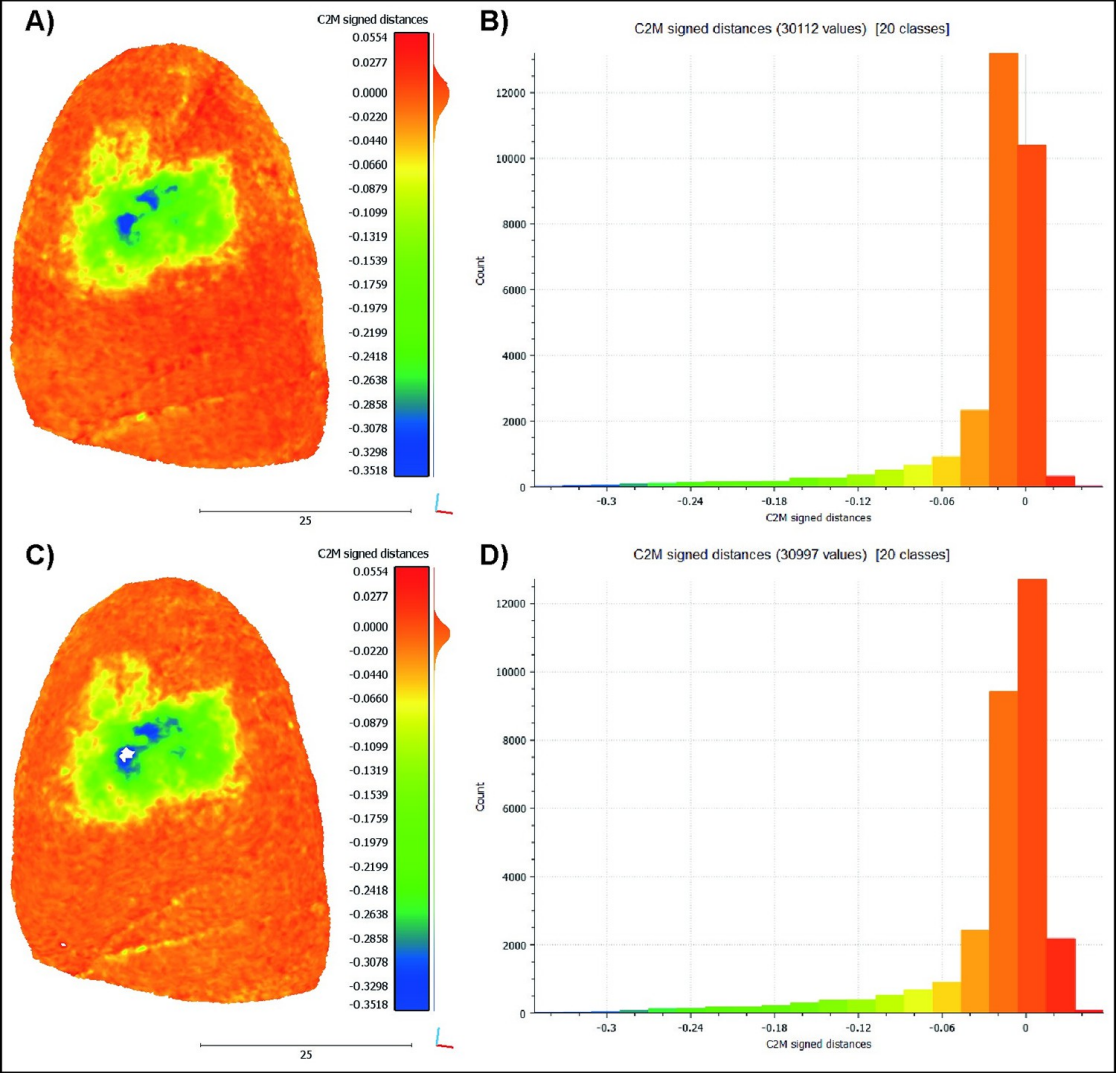
The analysis of GS18 uses surface. A) The T_0_ acquisitions 4 compared with T_4_ acquisitions 8, and its histogram B. C) The T_0_ acquisition 1 compared to T_4_ acquisition 1 and its histogram D.

However, in GS18 T_0_ acquisition 4 compared to T_4_ acquisition 8 (Fig 11), although the unused surface presents co-registration errors exceeding 100 μm in absolute value, the wear pattern is still visible and has not been affected by mesh distance errors. To confirm the precision of the analysis of the sole use surface, relevant areas were extracted from the models, excluding those that were not directly involved in the processing task. The same procedure was applied to T_0_ acquisition 1 compared to T_4_ acquisition 1 to contrast the outcome (Fig 12). The results are illustrated using false colour images indicating the distance between the reference item T_0_ models and the models of the tool at T_4_ (Fig 12A and 12C). The reference scale and the colour gradient ranges were different from the other analysis, to better describe the specific case. The reference scale spanning from 55.4 μm to -351.8 μm is appropriate to represent the distance between the use surface of GS18 T_0_ acquisitions 4 and the T_4_ acquisitions 8. However, applying the same scale to the use surface of GS18 T_0_ acquisition 1 compared to T_4_ acquisition 1 shows that the range is not enough to describe the distance between the use and unused surface. Moreover, from the histogram reported in Fig 12B and 12D, it is clear that the analysis performed on the data collected with the *ad hoc* setup resulted in a positive distance of about 21%, while in the other case, the percentage increases to 32%. As a result, it can be concluded that the strategy proposed in the literature underestimated the wear extension and resulted in incorrect mesh alignment with more surface having positive value.

**Fig 13:**
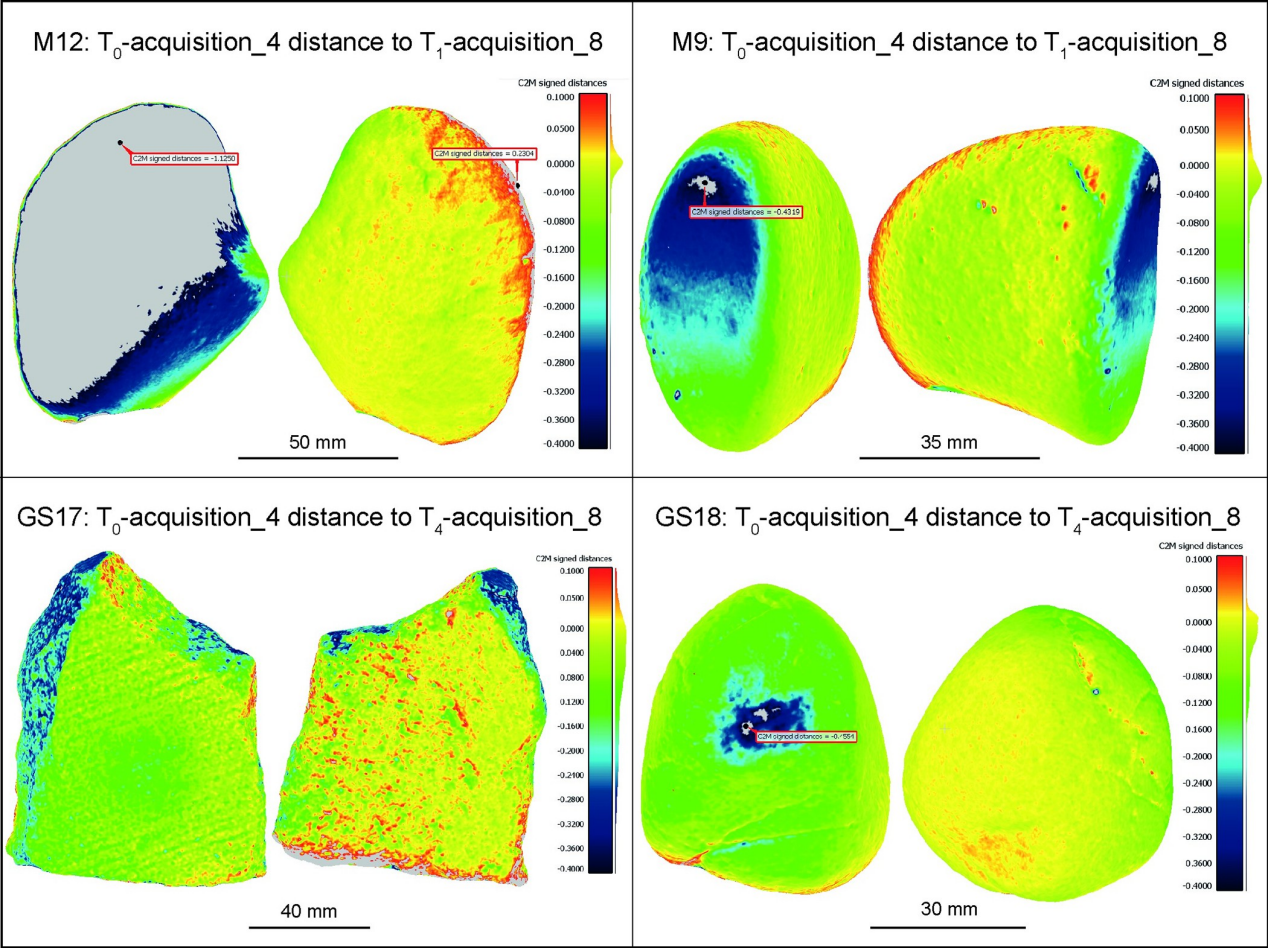
Acquisitions 4 of all GSTs at T_0_ compared with acquisitions 8 of the same GSTs at T_1_/T_4_ applying the ICP algorithm on the unused part of the tools. The process of importing the transformation matrices from the co-registration of T_0_ and T_1_/T_4_ half models into the T_1_/T_4_ entire models was carried out to determine if there would be an improvement in the mesh-to-mesh distance computation. However, the results obtained did not show any significant improvement over the previous test illustrated in Fig 11.

Also, the GST models acquired with the method available in the literature underwent a second test where only the unused half of the tool was co-registered with ICP before analysing the entire item (Fig 13). Although the results differ from those shown in Fig 11, no appreciable improvements are displayed. In fact, for GS18, which previously displayed the best results, the wear area extension is now visibly overestimated. The only exception is GS17, which, despite having significant co-registration errors especially at the stone edges, displays the morphology of the wear patterns in the middle of the use surface, although the deepness parameter is overestimated.

**Fig 14:**
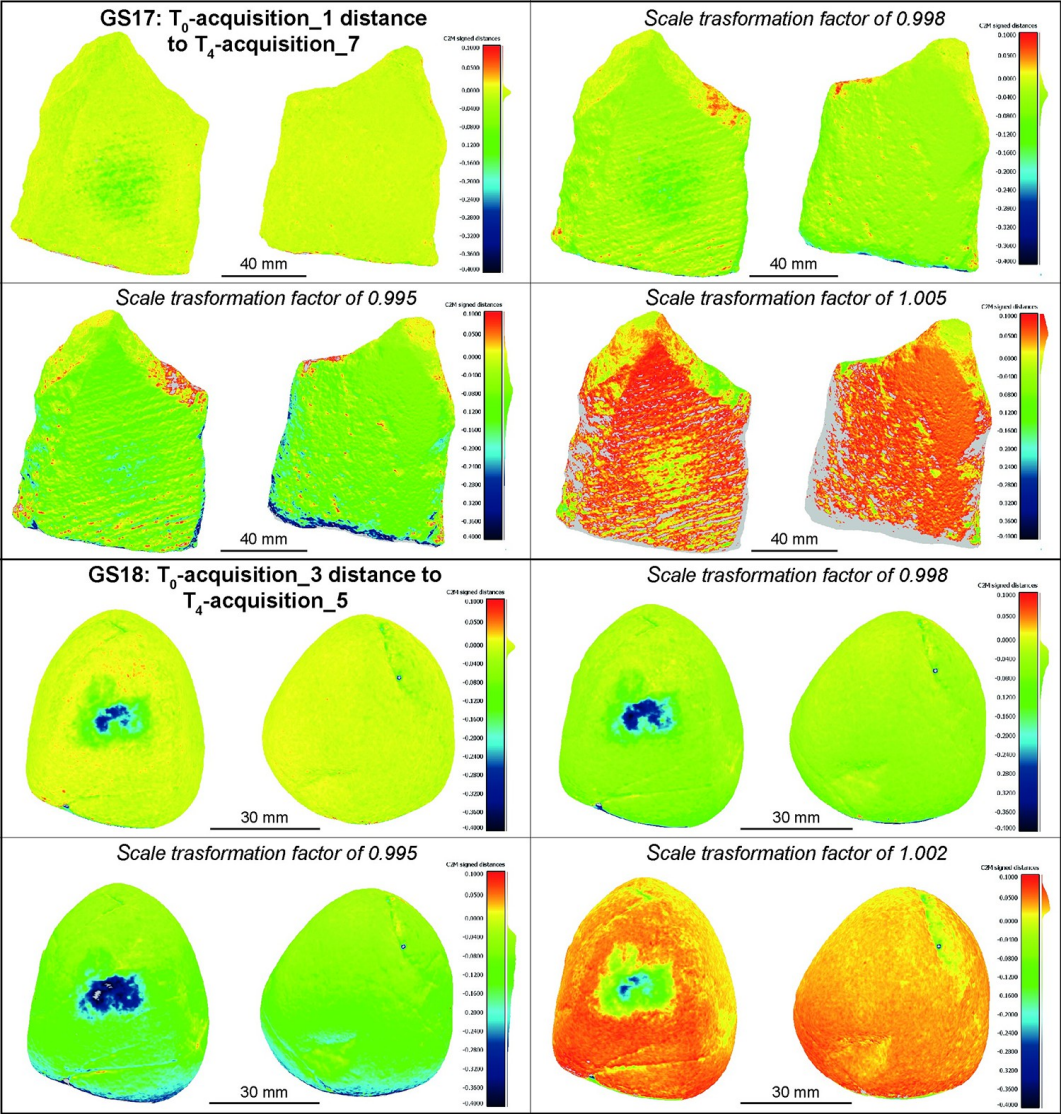
GS17 T_0_ acquisition 1 compared to T_4_ acquisition 7, and GS18 T_0_ acquisition 3 compared to T_4_ acquisition 5. Different scale factors were applied except on the first images.

As previously mentioned, every photogrammetric survey needs a proper scaling procedure. To create 3D models, a scale bar of a known length is included in the scene and used to scale the model. The end points of the scale bar are typically materialised by photogrammetric targets (i.e., circular coded, cross type) or by other signs (e.g. graduation of a ruler). However, the process of collimating these points on the scale bar during data processing, especially when done manually, can introduce human errors of several micrometres. As an example, Benito-Calvo and colleagues ([13]: 613) report a scale factor of 0.86 in their photogrammetric model compared to models acquired with a scanner. To demonstrate the impact of even smaller factors we performed 8 tests on GS17 and GS18 simulating a relative scaling error of 2‰ and 5‰, corresponding to a measurement error of ± 0.2 mm and ± 0.5 mm on a 100 mm scale bar. Models acquired at T_0_ were the reference items and kept unchanged while to the T_4_ models were applied 4 different scale factors: 0.998; 0.995; 1.002 and 1.005. Based on the analysis it is evident that even a few micrometres of errors when determining the scale factor can invalidate the results (Fig 14). In all the tests the co-registration errors were distributed across the entire surface of the objects, with errors within 100 μm in absolute value for the analysis using a scale factor of 0.998 and 1.002, but reaching up to approximately 450 μm in absolute value for those with a scale factor of 0.995 and 1.005. Additionally, the extension and depth of the wear trace patterns also changed, with noticeable differences already present in the models with a scale factor of 0.998 and 1.002, while the changes became dramatically evident in the case of 0.995 and 1.005.

## Discussion

Our experiments explore the application of close-range photogrammetry to trace and measure morphological changes in experimental ground stone tools at different stages of their replicative use. The analysis shows the depletion of stone surface, highlighting at the macroscale the extent of the contact area within the designated use surface during various stages of replicative use. Upon observing the samples under analysis, it was determined that active tools demonstrate more pronounced wear during achenes’ grinding activities compared to passive tools. However, the use-wear is localised to a smaller area of the surface. On the other hand, passive tools show use-wear over a larger area, but the depletion is less intense. The wear not only impacts the used surface but also extends to the unused surfaces, as indicated by the presence of cracks that deepen and extend, especially on the active tools. Furthermore, it is noteworthy that on the Moldovan stones, the damage appears to be more widespread across the use surface of both tools, whereas the depletion on the Italian pair of stones is more intense but concentrated in specific areas.

To reach our goals the adopted setup, the acquisition and the elaboration strategy were *ad hoc* calibrated to the study needs, and in particular to guarantee:

- stable and consistent acquisition settings, which also implied the use of a graded turntable, allowing for stable camera position;
- fixed camera parameters;
- homogeneous light even distributed across the object during acquisition;
- consistent scaling procedure;
- vertical and stable placement of the item allowed for the acquisition of the main faces of the object and its narrow parts in one rotation;
- narrow turntable rotation, and additional pictures taken to capture the thinner side of the object;
- the acquisition of the artefact without turning it to acquire the hidden side;
- acquisition environment that grants stable position and precise object repositioning;
- creation of a common file that is imported into all projects to ensure consistent camera calibration, item scaling, and orientation in space.

Previous studies have demonstrated that acquiring an object positioned vertically, with its main faces fully visible, along with the narrow lateral conjunction sides, enhances the overlap between sets of pictures and improves light-shadow stability. The improved overlap enhances the accuracy of the image orientation [9,24,34,40] and reduces the likelihood of gaps or multiple overlapping points in the resulting point-cloud. This phenomenon occurs particularly at the narrow edges of the object, where the conjunction of the two faces’ point-clouds occurs. This has a significant impact when studying stone assemblages, especially flaked tools, where diagnostic features are often located at the edges of the artefacts. To address this issue more effectively, we made the decision not to follow the conventional procedure of flipping the object 180 degrees to capture the hidden side. This decision has led to the exclusion of the portion of the stone attached to the pin from the acquisition. By excluding this non-diagnostic area, we prevent the merging of the two separate sets of pictures, thereby avoiding the potential issues mentioned earlier. Moreover, this refinement reduces the total number of required pictures, also optimising the time and PC performance required for the elaboration and eliminates the need for the time-consuming procedure of outlining the object’s silhouette (masking) to ensure picture orientation. In order to position the object vertically, other setup refinements have been proposed, such as using a nail/metal wire [33] or putty-like substance [24,33,34]. However, these methods are not feasible for heavy or geometrically complex objects where centering the barycenter is difficult. Based on previous studies recommendations [24,41], we opted to use a turntable to rotate the object to capture the different object’s angles so as to keep the camera steady and maintain consistent camera settings. The use of a wire or putty-like substance is not applicable when using a turntable, since the stability of the large items is not guaranteed during the rotation. In our experiment the choice to adopt metrological kinematic bases–permanently fixed to the objects and to the turntable–guarantees a stable position. Moreover, the kinematic bases ensure precise object repositioning in the acquisition environment, hence responding to another need of our research objectives. The impact of lighting was also addressed, as moving/adjusting the object position can significantly modify the appearance of uneven surfaces [34,39] thus creating problems with the orientation of the images. By avoiding flipping the object, we were able to mitigate this issue that was further minimised using a lightbox that homogeneously distributes the light coming from the LED panels on the item.

Consistent camera parameters and scaling factor–kept constant throughout all acquisition phases–revealed to be crucial factors, therefore camera automatic settings were avoided. To ensure internal consistency, a separate calibration file was imported into all item projects, and models were scaled using the 3D coordinates of the plate to ensure fidelity and consequently, comparability. Our study shows that even a relative scaling error of 2‰ can invalidate the analysis and lead to dramatically different results. This relative scaling error arises when the implemented procedures are not traceable. It is worth of note that in our work, a Brunson 803-MCL Length Reference Kit was used to ensure consistency and traceability of the photogrammetric measurements.

While our *ad hoc* setup involved an invasive procedure, making it applicable only to experimental tools, it was valuable in testing the limitations of literary-based setups and acquisition strategies, for the reasons mentioned earlier. However, when applying photogrammetry to archaeological tools, such invasive setups are not necessary because the focus is on observing the final modification induced by human activity, making comparisons irrelevant.

## Conclusion

Our preliminary study has successfully demonstrated the potential of close-range photogrammetry and of the implemented *ad hoc* setup in tracing and measuring morphological changes in experimental ground stone tools at various stages of their replicative use. Our approach provides a reference point for quantitative analysis and offers valuable insights for comparison with archaeological items. From a methodological perspective, our study, designed according to "preproducible" principles [27], emphasises the importance of carefully structuring an acquisition setup tailored to the study’s purpose. We also highlight the crucial need to (pre)determine the metrical requirements necessary to address specific research questions, while identifying and mitigating potential sources of errors that can arise from existing methods, thereby preventing misleading interpretations. To ensure highly consistent and repeatable results, we have developed a refinement of existing protocols that addresses potential sources of systematic errors. This process begins during the design stage of our experimental setup, allowing for the identification and mitigation of potential issues. However, it should be noted that for what concerns the implementation of the kinematic base, due to its invasive nature it is not suitable to be used with archaeological artefacts. Instead, our primary focus was on developing a reliable and accurate approach to measure wear patterns resulting from the tribological mechanisms during the use of experimental GSTs considering the tool’s entire geometry. This approach enables the creation of a proxy to be applied for comparison when inferring the intentional use of the archaeological artefacts. In the archaeological context, we only have access to one step of tool usage without information about the other stages along the functional biography of the stone tools. Therefore, the models generated from our sequential replicative experiments play a pivotal role in enhancing our comprehensive understanding of the archaeological evidence. They serve as a valuable resource for approximating the missing stages of tool usage and contribute to a more accurate interpretation of the archaeological record.

## References

[1] Adams JL. Ground Stone Analysis: a technological approach. The University of Utah Press; 2002.

[2] Semenov SA. Prehistoric Technology. 1st ed. New York: Barnes and Noble; 1964.

[3] Tringham R, Cooper G, Odell G, Voytek B, Whitman A. Experimentation in the formation of edge damage: a new approach to lithic analysis J. Field Archaeol. 1974; 1: 171–96.

[4] Odell GH. The application of micro-wear analysis to the lithic component of an entire prehistoric settlement: methods, problems, and functional reconstructions. PhD Thesis, Harvard University, Cambridge; 1977.

[5] Hayden B. Lithic Use-Wear Analysis. New York: Academic; 1979.

[6] Keeley LH. Experimental Determination of Stone Tool Uses: A Microwear Analysis. Chicago: University of Chicago Press; 1980.

[7] Wyatt-Spratt S. After the revolution: a review of 3D modelling as a tool for stone artefact analysis. JCAA. 2022; 5(1): 215–237.

[8] Shott M. Digitalizing archaeology: a subtle revolution in analysis. World Archaeol. 2014; 46(1): 1–9.

[9] Magnani M, Douglass M, Schroder W, Reeves J, Braun DR. The digital revolution to come: photogrammetry in archaeological practice. Am. Antiq. 2020; 85(4): 737–760.

[10] Caruana MV, Carvalho S, Braun DR, Presnyakova D, Haslam M, Archer W, et al. Quantifying traces of tool use: a novel morphometric analysis of damage patterns on percussive tools. PLoS ONE. 2014; 9(11): e113856. doi: 10.1371/journal.pone.0113856 25415303 PMC4240665

[11] Benito-Calvo A, Carvalho S, Arroyo A, Matsuzawa T, de la Torre I. First GIS analysis of modern stone tools used by wild chimpanzees (*Pan troglodytes verus*) in Bossou, Guinea, West Africa. PLoS ONE. 2015; 10(3): e0121613. 25793642 10.1371/journal.pone.0121613 PMC4368754

[12] Benito-Calvo A, Arroyo A, Sánchez-Romero L, Pante M, de la Torre I. Quantifying 3D Micro-Surface Changes on Experimental Stones Used to Break Bones and Their Implications for the Analysis of Early Stone Age Pounding Tools. Archaeometry. 2017; 60: 419–436.

[13] Benito-Calvo A, Crittenden AN, Livengood SV, Sánchez-Romero L, Martínez-Fernández A, de la Torre I, Pante M. 3D 360° surface morphometric analysis of pounding stone tools used by Hadza foragers of Tanzania: a new methodological approach for studying percussive stone artefacts. J. Archaeol. Sci. Rep. 2018; 20: 611–621.

[14] Caricola I, Zupancich A, Moscone D, Mutri G, Falcucci A, Duches R, et al. An integrated method for understanding the function of macro-lithic tools. Use wear, 3D and spatial analyses of an Early Upper Palaeolithic assemblage from North Eastern Italy. PLoS ONE. 2018; 13(12): 1–46 doi: 10.1371/journal.pone.0207773 30540784 PMC6291187

[15] Zupancich A, Mutri G, Caricola I, Carra ML, Radini A, Cristiani E. The application of 3D modeling and spatial analysis in the study of groundstones used in wild plants processing. Archaeol Anthropol Sci. 2019; 11: 4801–4827.

[16] Arroyo A, de la Torre I. Pitted stones in the Acheulean from Olduvai Gorge Beds III and IV (Tanzania): A use-wear and 3D approach. J. Hum. Evol. 2020; 145: 102837. doi: 10.1016/j.jhevol.2020.102837 32652256

[17] Zupancich A, Cristiani E. Functional analysis of sandstone ground stone tools: arguments for a qualitative and quantitative synergetic approach. Sci. Rep. 2020; 10(1): 1–13. 32978414 10.1038/s41598-020-72276-0 PMC7519652

[18] Longo L, Skakun NN, Pantyukhina IE, Terekhina VV, Sorrentino G. Aurignacian grinding stone from Surein I (Crimea): “trace-ing” the roots of starch-based diet. J. Archaeol. Sci.Rep. 2021; 38: 102999.

[19] Hayes EH, Field JH, Coster ACF, Fullagar R, Matheson C, Florin SA, et al. Holocene grinding stones at Madjedbebe reveal the processing of starchy plant taxa and animal tissue. J. Archaeol. Sci. Rep. 2021; 35: 102754.

[20] Hayes EH, Fullagar R, Field JH, Coster ACF, Matheson C, Nango M, et al. 65,000-years of continuous grinding stone use at Madjedbebe, Northern Australia. Sci. Rep. 2022; 12: 11747. doi: 10.1038/s41598-022-15174-x 35817808 PMC9273753

[21] Paixão E, Pedergnana A, Marreiros J, Dubreuil L, Prévost M, Zaidner Y, et al. Using mechanical experiments to study ground stone tool use: Exploring the formation of percussive and grinding wear traces on limestone tools. J. Archaeol. Sci. Rep., 2021; 37: 102971.

[22] Paixão E, Marreiros J, Dubreuil L, Gneisinger W, Carver G, Prévost M, et al. Middle Paleolithic ground stones tools of Nesher Ramla unit V (Southern Levant): A multi-scale use-wear approach for assessing the assemblage functional variability. Quat. Int. 2022; 624: 94–106.

[23] Sorrentino G, Longo L, Obada T, Borghi A, Re A, Paggi M, et al. Tracing old gestures: a multiscale analysis of ground stone tools developed on sequential lab-controlled replicative experiments. Heritage 2023; 6(6): 4737–4767.

[24] Porter ST, Roussel M, Soressi M. A simple photogrammetry rig for the reliable creation of 3D artifact models in the field. Adv. Archaeol. 2016; 4(1): 71–86.

[25] Green S, Bevan A, Shapland M. A comparative assessment of structure from motion methods for archaeological research. J. Archaeol. Sci. 2014; 46: 173–181.

[26] Rilievo Barone S. 3D a luce strutturata. In: Siano S., editor. Archeometria e restauro. L’innovazione tecnologica. Nardini editore; 2012; p. 19–26 (Italian).

[27] Stark PB. Before reproducibility must come preproducibility. Nature. 2018; 557: 613. doi: 10.1038/d41586-018-05256-0 29795524

[28] Allsworth-Jones P, Borziac IA, Chetraru NA, French CAI, Medyanik SI. Brînzeni: A Multidisciplinary Study of an Upper Palaeolithic site in Moldova. Proceedings of the Prehistoric Society. 2018; 84: 41–76.

[29] Sorrentino G, Lo Giudice A, Re A, Borghi A, Longo L. Più di un semplice ciottolo: Un protocollo sperimentale per la comprensione del ruolo di strumenti macrolitici nella trasformazione delle risorse vegetali nel Paleolitico Superiore in Eurasia. Archeologie sperimentali. Temi, metodi, ricerche. 2021; 2: 24–40 (Italian).

[30] Hardy BL. Climatic variability and plant food distribution in Pleistocene Europe: implications for Neanderthals diet and subsistence. Quant. Sci. Rev. 2010; 29: 662–679.

[31] Longo L, Altieri S, Birarda G, Cagnato C, Cefarin N, Graziani V, et al. A Multi-Dimensional Approach to Investigate Use-Related Biogenic Residues on Palaeolithic Ground Stone Tools. Environ. Archaeol. 2021: 1–29.

[32] Longo L, Birarda G, Cagnato C, Badetti E, Covalenco S, Pantyukhina I, et al. Coupling the beams: How controlled extraction methods and FTIR-spectroscopy, OM and SEM reveal the grinding of starchy plants in the Pontic steppe 36,000 years ago. J. Archaeol. Sci. Rep. 2022; 41: 103333.

[33] Magnani M, Douglass M, Porter ST. Closing the seams: resolving frequently encountered issues in photogrammetric modelling. Antiquity. 2016, 90(354): 1654–1669.

[34] Sapirstein P. A high-precision photogrammetric recording system for small artifacts. J. Cult. Herit. 2018; 31: 33–45.

[35] ThorLabs. Kinematic Bases [Cited 2023 May 15]. Available from: https://www.thorlabs.com/NewGroupPage9_PF.cfm?Guide=10&Category_ID=13&ObjectGroup_ID=1546.

[36] Brunson. Scale Bar for laser trackers [Cited 2023 May 15]. Available from: https://www.brunson.us/products/laser-tracker-products/metrology-3d-measurement-smr-target-holders-scale-bars.html.

[37] Fraser CS. Digital camera self-calibration. ISPRS Journal of Photogrammetry and Remote sensing. 1997; 52(4): 149–159.

[38] Adams JL, Delgado-Raack S, Dubreuil L, Hamon C, Plisson H, Risch R. Functional analysis of macro-lithic artefacts: a focus on working surface. In: Sternke F, Eigeland L, Costa LJ, editors. Non-flint raw material use in prehistory old prejudices and new direction. Proceedings of UISPP congress, Lisbon, Portugal, September 2006, BAR-IS, 2009, pp. 43–66.

[39] Karami A, Menna F, Remondino F, Varshosaz M. Exploiting light directionality for image‐based 3d reconstruction of non‐collaborative surfaces. The Photogrammetric Record. 2022; 37(177): 111–138.

[40] Antinozzi S, Fiorillo F, Surdi M. Cuneiform Tablets Micro-Surveying in an Optimized Photogrammetric Configuration. Heritage. 2022; 5(4): 3133–3164.

[41] Nicolae C, Nocerino E, Menna F, Remondino F. Photogrammetry applied to problematic artefacts. In: Remondino F, Menna F, editors. ISPRS Archives XL-5; 2014: 451–456.

